# Intracellular Quantum Sensing of Free-Radical Generation Induced by Acetaminophen (APAP) in the Cytosol, in Mitochondria and the Nucleus of Macrophages

**DOI:** 10.1021/acssensors.2c01272

**Published:** 2022-11-10

**Authors:** Rokshana Sharmin, Anggrek C. Nusantara, Linyan Nie, Kaiqi Wu, Arturo Elias Llumbet, Willem Woudstra, Aldona Mzyk, Romana Schirhagl

**Affiliations:** † University Medical Center Groningen, Department Biomedical Engineering, Groningen University, Antonius Deusinglaan 1, 9713 AV Groningen, The Netherlands; ‡ Laboratory of Genomic of Germ Cells, Biomedical Sciences Institute, Faculty of Medicine, University of Chile, Independencia, 1027 Independencia Santiago, Chile; § Institute of Metallurgy and Materials Science, Polish Academy of Sciences, Reymonta 25, 30-059 Krakow, Poland

**Keywords:** NV centers, relaxometry, nanodiamonds, cells, liver toxicity

## Abstract

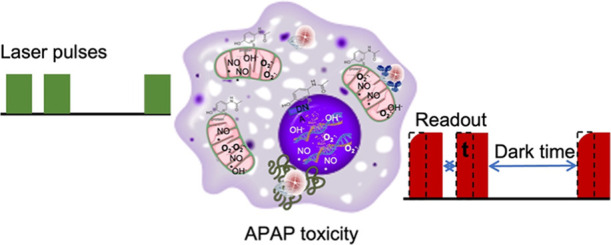

Acetaminophen overdoses cause cell injury in the liver. It is widely accepted that liver toxicity is initiated by the reactive *N*-acetyl-*para*-aminophenol (APAP) metabolite *N*-acetyl-*p*-benzoquinone imine (NAPQI), which first depletes glutathione and then irreversibly binds to mitochondrial proteins and nuclear DNA. As a consequence, mitochondrial respiration is inhibited, and DNA strands break. NAPQI also promotes the oxidative stress since glutathione is one of the main free-radical scavengers in the cell. However, so far it is unknown where exactly free radicals are generated. In this study, we used relaxometry, a novel technique that allows nanoscale magnetic resonance imaging detection of free radicals. The method is based on fluorescent nanodiamonds, which change their optical properties based on their magnetic surrounding. To achieve subcellular resolution, these nanodiamonds were targeted to cellular locations, that is, the cytoplasm, mitochondria, and the nucleus. Since relaxometry is sensitive to spin noise from radicals, we were able to measure the radical load in these different organelles. For the first time, we measured APAP-induced free-radical production in an organelle-specific manner, which helps predict and better understand cellular toxicity.

Acetaminophen [*N*-acetyl-*para*-aminophenol (APAP)] is the most used analgesic and antipyretic drug. Overdose of APAP can lead to acute liver poisoning and death.^1,2^ Physiologically, APAP is metabolized in the liver, by cytochrome P450s,^3−5^ which produces the active metabolite *N*-acetyl-*p*-benzoquinone imine (NAPQI) that is efficiently detoxified by conjugation with glutathione.^6,7^ However, higher doses of APAP (0.5 to 10 mM/mL) lead to the critical depletion of glutathione, formation of NAPQI adducts with mitochondrial proteins, and oxidative stress. This oxidative stress amplifies the formation of reactive oxygen species (ROS) and reactive nitrogen species, causing mitochondrial membrane permeability transition, pore opening, and cessation of adenosine triphosphate synthesis. In addition, mitochondrial matrix swelling ruptures the outer membrane and releases endonucleases, which cause nuclear deoxyribonucleic acid (DNA) fragmentation. Together, the nuclear DNA damage and the extensive mitochondrial dysfunction result in necrotic cell death.^8^

Here, we investigated the effect of APAP on macrophages since the response of macrophages to APAP toxicity is highly relevant for liver toxicity and controversial. The liver contains the largest proportion of macrophages among all solid organs in the body.^9^ Every 100 liver cells are accompanied by 20–40 macrophage cells in the liver.^10^ Liver macrophages perform crucial functions to maintain homeostasis for the liver itself and for the whole body. They scavenge bacteria and microbial products that come to liver from the intestine via the portal vein, sense disturbances in tissue integrity, and serve as a gatekeeper for initiating or suppressing immune responses.^11^ At the same time, they contribute to the progression of liver diseases, including hepatitis, fibrosis, and cancer.^12^ Overdose of APAP produces ROS in macrophages and damages the mitochondria, nucleus, and lipids. As a result, cells die and cause liver toxicity.^13^ However, hepatic macrophages also play a hepato-protective role through the production of cytokines and mediators, such as IL-10, IL-6, and IL-18-binding proteins, which counteract inflammatory events and promote liver regeneration.^14^

There are many methods to detect cellular toxicity, including imaging, cell loss, monitoring of DNA damage, measurement of the reduction of glutathione levels, and detection of intracellular ROS and mitochondrial membrane potential.^15^ The intracellular concentration of reactive species is one of the most important indicators of liver toxicity. However, most methods do not differentiate between the different types of reactive species. Among the reactive species, free radicals such as hydroxyl radical, nitric oxide, or superoxide are the most difficult to measure due to their low abundance and reactivity.^16^ Free radicals are responsible for cellular damage by reacting with cellular components such as proteins, lipids, and DNA.^17^ Therefore, it is very important to carry out reliable measurements of the concentration or relative levels of free radicals in addition to the conventional measurements of ROS. Several methods are available for free radical detection. Chromatographic methods are used for the separation and identification of adducts or reaction products that free radicals produce with other molecules. Spectrophotometric methods are based on the reaction between radicals and redox substances; the resulting differences in absorbance at different wavelengths give semi-quantitative data of free radical levels. However, these methods do not offer any spatial resolution.

Fluorescent and chemiluminescent probes and electron spin resonance (ESR/EPR) offer at least some spatial resolution, but these techniques do not allow subcellular resolution due to the diffusion of dye molecules.^18^ For example, 2′,7′-dichlorofluorescin diacetate (DCFH-DA) is a probe generally used for the direct measurement of intracellular ROS including all kinds of radicals and non-radical species.^19,20^ However, because non-radical species are in a much higher concentration than radicals, these are the main components contributing to the detected signal.

Diamond-based relaxometry is an appealing option to solve the spatial limitations of the aforementioned methods since it can detect the sum of all free radicals in the sensing volume. The technique allows sensing at a nanomolar range with nanoscale resolution. The method requires the use of fluorescent nanodiamonds (FNDs). These are promising nanoprobes, for their stable fluorescence and excellent biocompatibility.^21^ They have unique magneto-optical properties and can be used as probes for magnetic resonances^22,23^ as well as pressure^24−26^ and temperature measurements.^27^ Due to their unprecedented sensitivity, FNDs can detect even the faint magnetic resonance of a single electron or even a few nuclear spins.^28^ Since these signals are strongly distance dependent, FNDs only sense their immediate surrounding up to a few nanometers. As a result, the diamond probes have to be close to the molecules that need to be detected. Our group already proved that FNDs can be used to detect free radicals in living cells.^29,30^ Since their free electrons cause spin noise, they can be detected with relaxometry measurements (also called T1 measurements). This is a specific mode of diamond magnetometry that is purely optical and does not require microwaves.^31^ With this method, radicals can be detected with nanoscale resolution in nanomolar concentrations.

In this study, our aim was to predict APAP-induced cellular toxicity at an early stage before cell death by measuring free radical concentrations. For the first time, we achieved spatial resolution, which allowed differentiating between radical generation in the cytosol, the mitochondria, and the nucleus. To achieve this goal, we used diamond-based quantum sensing and compared with conventional methods.

## Materials and Methods

### Cell Experiments

Macrophage J774A.1 cells were grown in high glucose Dulbecco’s modified Eagle’s medium (DMEM) containing 10% (v/v) fetal bovine serum. This medium was supplemented with 1% streptomycin and 1% penicillin. Cells were grown in T-75 cell culture flasks containing (0.5–1) × 10^6^ cells/mL and kept in a humidified atmosphere containing 5% CO_2_ at 37 °C. On the following day, when the number of cells reached 5 × 10^6^ cells/mL, cells were removed from the T-75 flask and seeded in a four quarters glass bottom Petri dish (Greiner Bio-One, Germany) at 30,000 cells/cm^2^ with 500 μL of DMEM medium and kept in the incubator at 37 °C in the presence of 5% CO_2_.

The FND incubation process in macrophage cells was described in our previous paper.^21^ For measurements in cells, 1 μg/mL suspensions of bare FNDs, FNDs targeted to mitochondria (MIT-FNDs) and to the nucleus (NLS-FNDs) were prepared with high glucose DMEM medium for targeting cytoplasm, mitochondria, and nucleus, respectively. Then, cells were treated with different concentrations of APAP varying the incubation time.

In the case of 18 h treatment, we incubated different targeting nanoparticles 2 h before the treatment. In the 3, 6, and 9 h groups, different targeting FNDs were incubated overnight without treatment. Next morning, we added differently concentrated APAP solutions with different concentrations to the DMEM medium. Then, relaxometry assays were performed to determine the different stress responses. After that, cells were fixed with 4% formaldehyde since in fixed cells where particles remain at a specific place, better image quality can be obtained. After fixation, cells were washed with phosphate buffer saline before staining with 4′,6-diamidino-2-phenylindole (DAPI) and fluorescein (FITC)-phalloidin, as previously described^32^ to visualize nuclei and F-actin, respectively. For staining, 1% bovine serum albumin (BSA) was added with phosphate-buffered saline (PBS) to prepare PBSA solution. Then, cell samples were washed with PBSA 3 times. Subsequently, we added the staining solution prepared by mixing 2 μg/mL FITC and 4 μg/mL DAPI in the PBSA solution. Then, samples were wrapped with aluminum foil and shaken for 1 h for staining. After staining, we washed the cells three times with PBSA and PBS again. Finally, 500 μL of PBS was added to store the sample for imaging.

### Preparation of FNDs

To determine the T1 relaxation time, we used 70 nm FNDs (Adamas Nanotechnologies, NC, USA). These FNDs are produced by high-pressure high-temperature synthesis followed by irradiation and high-temperature annealing. This process leads to bright particles containing about 300 nitrogen vacancy (NV^–^) centers per particles (determined by the manufacturer). As a last step of production, FNDs are cleaned with oxidizing acids. As a result, they are oxygen terminated. These particles are widely used for different applications and have been characterized in the literature.^33−35^

**Figure 1:**
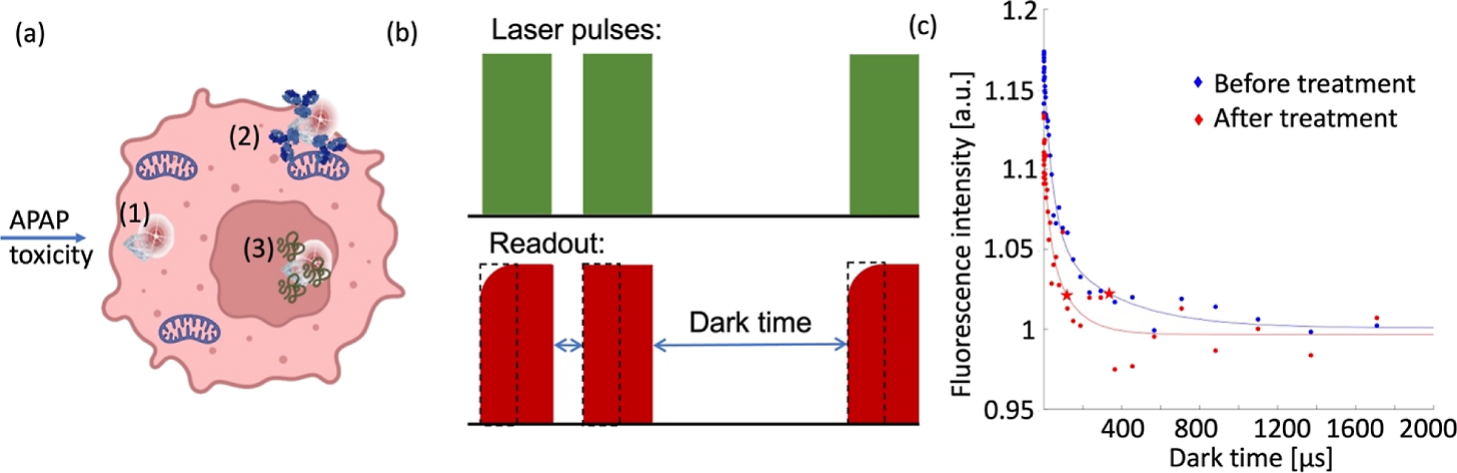
Experiments conducted in this paper. (a) Schematic representation of the conducted experiments. Three different particles were used for sensing free radical generation in macrophage J774A.1 cells. (1) Bare FNDs, which allow measurements in the cytosol, (2) FNDs coated with an antibody targeting the mitochondrial surface (MIT-FNDs), and (3) FNDs coated with a nuclear localization signal (NLS-FNDs). (b) Schematic representation of the relaxometry sequence which is used to measure the spin noise from free radicals. The laser pulses pump the NV centers in the bright *m*_s_ = 0 state of the ground state. After different dark times, another laser pulse probes if the NV centers are still in the bright state or have returned to the darker equilibrium. The dotted rectangles indicate the read window. (c) Example of two representative T1 curves measured in macrophage J774A.1 cells before and after they were challenged with APAP for 18 h. Red stars indicate the T1 time.

In this article, we used three types of particles, which are shown in Figure 1a: bare FNDs for measurements in the cytoplasm, particles for mitochondrial targeting (MIT-FND), and particles for nucleus targeting (NLS-FND).

#### FNDs for Cytoplasmic Measurements

For cytoplasmic measurements, a suspension of bare FNDs (1 μg/mL) was prepared in high glucose DMEM medium. Then, the solution was added to the cells. The solution containing FNDs remained in the dish until the end of the experiment. To further confirm the location of the particles, we performed calcein assay (see Supporting Information Figure S5). The assay was performed in the presence of different concentrations of APAP, and we added 0.25 mM calcein with 1 μg/mL FNDs. After 2 h of FNDs and calcein incubation, we added APAP for about 18 h. At the end of the treatment, the cells were washed three times with PBS and fixed with 4% formaldehyde before taking confocal images. The colocalization of FNDs with the vesicles was quantified with the Manders’ coefficient using the Fiji plugin “JACop”.^36,37^

#### FNDs for Mitochondrial Measurements

Anti-VDAC2 antibody (GTX104745, GeneTex, The Netherlands) coating the FNDs were targeted to the surface of mitochondria. To prepare these particles, anti-VDAC2 antibodies were diluted to a concentration of 0.089 mg/mL (1:100 dilution, as suggested by the manufacturer). The antibodies were mixed with FNDs (1 μg/mL) in a 1:2 ratio for 1 to 2 min while vortexing. This was followed by incubation at room temperature for 15 min to allow antibodies to adsorb on the FNDs. Malvern instruments (dynamic light scattering, Malvern Instruments Ltd, Malvern, UK) were used to evaluate aggregation in medium after the modification of nanodiamonds to exclude aggregates of the coated FNDs (1 μg/mL) in medium (see Supporting Information Figure S1).

#### FNDs for Nuclear Measurements

For attaching NLS to the FNDs, we used 1-ethyl-3-(3-dimethylaminopropyl)-carbodiimide hydrochloride (EDC, Merck, The Netherlands) and *N*-hydroxysuccinimide (NHS, Merck, The Netherlands). The resulting surface groups were reacted with amine groups of SV40 T-Ag-derived NLS peptide (PKKKRKVEDPYC) (AnaSpec-AS63788) to form carbamide. Specifically, 0.5 mL of freshly prepared EDC/NHS (30/15 μg·mL^–1^, respectively) solution made with cold ultrapure water was added to 200 μg FND in 1 mL of ultrapure water. The reaction was stirred for 1 h in ice to activate the carboxyl groups on the nanodiamond surface. Then, 50 μg of NLS peptide in 0.5 mL of ultrapure water was added to the activated FND solution. The reaction was performed overnight at room temperature. Unconjugated NLS peptides and the residues of EDC/NHS were removed by dialysis against (Spectra/Pore, MWCO 10,000–16,000) demi water. After purification, NLS-modified FNDs (NLS-FND) were concentrated by PEG (MW: 8000 Da, Merck, The Netherlands). The concentration was achieved by placing NLS-FNDs in a dialysis bag (MWCO: 3500 Da). Then, the dialysis bag was buried in PEG powder (MW: 8000 Da, Merck, The Netherlands). This way, water is removed from the dialysis bag, while PEG cannot enter that bag. After that the material was sterilized using sonication. NLS-FNDs were sonicated 10 to 15 min before mixing with high glucose DMEM cell culture medium. A Malvern Zetasizer nanosystem (dynamic light scattering, Malvern Instruments Ltd, Malvern, UK) was used to evaluate size changes after the modification of nanodiamonds to exclude severe aggregation of the coated FNDs (1 μg/mL) in medium (see Supporting Information Figure S1).

To determine whether NLS-FNDs are on the nucleus, we first incubated cells with NLS-FNDs, and then cells were washed with PBS three times and fixed with 4% formaldehyde. Then, cells were washed again three times with PBS, and we stained the nuclei with 4 μg/mL DAPI, as previously demonstrated by Hemelaar et al.^32^ To confirm that NLS-FND colocalizes with the nucleus, we used Manders’ coefficients, as described above.

#### Evaluation of Spin Sensing Capacity of Antibody Coated and NLS Conjugated FNDs

To evaluate the spin sensing capacity of bare-FNDs, MIT-FNDs, and NLS-FNDs, we measured T1 relaxation times of these particles in the presence of different concentrations of GdCl_3_ solution in DMEM medium. Gadolinium strongly reduces T1 and is thus used as a contrast agent in magnetic resonance imaging (MRI). For all particles, we were able to confirm that they respond to spin noise by lowering T1 as expected. Before performing the experiment, we instantly prepared different gadolinium stock solutions (1, 10, 100, and 1000 μM) by dissolving gadolinium(III) chloride (439770-5G, Sigma-Aldrich, Germany) in MQ water. During the experiments, we added 5 μL from each stock solution to generate the desired 0.01, 0.1, 1, and 10 μM working concentrations.

On the other hand, we coated Petri dishes with bare-FNDs, MIT-FNDs, and NLS-FNDs inside the flow hood. There, 1 μL of bare FNDs, MIT-FNDs, or NLS-FND (1 μg/mL) suspension were spread on different glass bottom Petri dishes and continuously spreading with tips to avoid aggregation while drying. Then, the Petri dish was focused on the confocal plane of a home-built confocal microscope.^38^ We scanned the Petri dish to select single FND and added 500 μL DMEM medium and measured T1. Subsequently, we added 0.01, 0.1, 1, and 10 μM GdCl_3_ solution and measured T1 (see Supporting Information Figure S2).

### Relaxometry Equipment

For the experiments in this article, we used equipment that is similar to what is used in the field and which has been reported before.^38,39^ In short, the setup is a confocal microscope with the capability to perform the required pulsing sequences (as shown in Figure 1b). For pulsing, the laser is directed through a beam splitter cube which directs the beam through an acoustooptical modulator, through a λ/4 plate, and is then reflected back through the same aperture, which allows fast and precise laser pulsing. Detection is carried out via an avalanche photodiode (Excelitas, SPCM-AQRH) as a detector The setup is equipped with a green Neodym YAG laser at 532 nm, and we have the ability to track particles in 3D. The sample stage is designed in a way that allows for standard glass-bottom Petri dishes to be measured. For identification of cells, we used a bright-field microscope to collect images simultaneously. Bright-field illumination was achieved with a 470 nm fiber-coupled LED supplied with T-Cube LED driver. The images were acquired with a Compact USB 2.0 CMOS Camera from Thorlabs and an Olympus PLN 4× objective to focus the blue light with NA 0.1. To avoid damage to the cells by light, we used a relatively low laser power of 50 μW (measured at the sample position in continuous illumination). To tune laser intensities, the beam is directed through a manually adjustable filter wheel.

### Relaxometry

After completion of the desired time of drug treatment with APAP, the Petri dish was focused at the focal point of a home-built magnetometry setup that has been explained previously.^40^ To perform the T1 measurement, a single FND inside the cell (confirmed by confocal imaging) was selected. We chose particles with a count rate between 1 × 10^6^ and 3 × 10^6^ photon counts per second. Particles with larger count rates are usually large aggregates, while smaller count rates indicate a small particle. Such small particles move faster and emit less photons per time unit and thus deliver less reproducible data. During a relaxation time measurement, the NV centers are first initialized into the bright *m*_s_ = 0 state of the ground state by a laser pulse (5 μs). Then, we probed after varying dark times if they are still in this state or if they have returned into the darker equilibrium between the *m*_s_ = 0 and *m*_s_ = +1 or −1 states. This is carried out by counting photons in the first 0.490 μs of the pulse (see Figure 1b). Lower photon counts indicate that the NV centers have already returned to the equilibrium. The time it takes the NV centers to relax into the equilibrium, the T1 time, decreases in the presence of spin noise. While an individual T1 measurement takes on the order of microseconds, we repeated the pulse train for 10,000 times to improve the signal-to-noise ratio. The entire experiment lasts around 10 min, and each experiment was repeated three times independently.

### DCFH-DA Assay

Intracellular ROS were measured using the cell permeable probe DCFH-DA, which measures the total intracellular ROS that was produced between adding the compound and detection. After entering the cell, DCFH-DA was deacetylated and later oxidized by ROS to 2′,7′-dichlorodihydro fluorescein (DCF) which is fluorescent. After APAP treatment, cells were incubated with 0.2 μg/mL of a 5 μM DCFH-DA solution for 30 min at 37 °C. Subsequently cells were washed with PBS, and we immediately acquired 2D confocal images with a Zeiss 780 confocal microscope (Zeiss, Sliedrecht, The Netherlands), using excitation and emission wavelengths of 485 and 582 nm, respectively. The mean fluorescence intensity was measured using Fiji software. Each experiment was repeated three times, and the number of cells per experiment was more than 50.

### DHE Assay

Dihydroethidium (DHE) itself displays blue fluorescence in the cell cytoplasm. However, the oxidized form 2-hydroxyethidium intercalates into DNA and exhibits red fluorescence. DHE (abcam) was used to determine the intracellular superoxide radical levels qualitatively.^41^ DHE enters the cells and is oxidized to ethidium, which binds to DNA to produce red fluorescence. In this study, a DHE solution (2 μg/mL) was prepared in DMEM medium and added to macrophage cells immediately after completing the APAP treatment. Then, the cells were incubated for 10 min at 37 °C and 5% CO_2_ in the incubator. Subsequently cells were washed with ice-cold DMEM medium, and 2D confocal images were taken with a Zeiss 780 confocal microscope (Zeiss, Sliedrecht, The Netherlands). DHE excitation and emission wavelengths are 514 and 580 nm, respectively. The mean fluorescence intensity was quantified using FIJI software. Each experiment was repeated three times, and the number of cells per experiment was more than 50.

### MTT Assay

The 3-(4,5-dimethylthiazol-2-yl)-2,5-diphenyltetrazolium bromide (MTT) assay is used to measure cellular metabolic activity as an indicator of cell viability, proliferation, and cytotoxicity. This colorimetric assay is based on the reduction of a yellow tetrazolium salt (MTT, Sigma-Aldrich, Zwijndrecht, the Netherlands) to purple formazan crystals by metabolically active cells. Viable cells contain NAD(P)H-dependent oxidoreductase enzymes that reduce the MTT to formazan. The insoluble formazan crystals are dissolved using a solubilization solution. The resulting, colored solution is quantified by measuring absorbance at 570–650 nanometers using a multi-well spectrophotometer. The darker the solution, the higher the number of viable metabolically active cells. To test the viability of cells after APAP treatment, cells were treated with 0.05% MTT and serum-free medium for 2 h. After 2 h of incubation, the cells were washed with PBS. Subsequently, formazan crystals were dissolved using 2-propanol, and the absorption of the purple solution was measured using a Synergy HT microplate reader, Biospx, USA at 570 to 650 nm.

### Statistical Analysis

Statistical analysis of all data was conducted by using GraphPad Prism version 6. Significance was tested by using a two-way ANOVA followed by a Tukey post hoc test. All statistical tests were compared to the control group and defined as: ns *P* > 0.05, *P ≤ 0.05, **P ≤ 0.01, ***P ≤ 0.001, and ****P ≤ 0.0001.

## Results and Discussion

### Confirming the Subcellular Location of Diamonds

For bare FNDs, it has been shown that they escape from the endosomes and reside in the cytosol after entering the cell.^42,43^ There are substantial differences in endosomal escape between cell types.^44^ Endosomal escape has also been shown for macrophage J774A.1 cells in the Figure S5. Calcein assay which we used here is widely used to evaluate the presence of particles inside the cytoplasm. Calcein is a cell membrane impermeant fluorescent dye and emits green light in endosomes and lysosomes. If particles escape the endosome, they do not colocalized with green vesicles. Although both antibody targeting and targeting with NLS are established for other particles, it is important to confirm their subcellular location. For mitochondrial targeting with MIT-FND, this has been confirmed for this cell type in ref (30).

**Figure 2:**
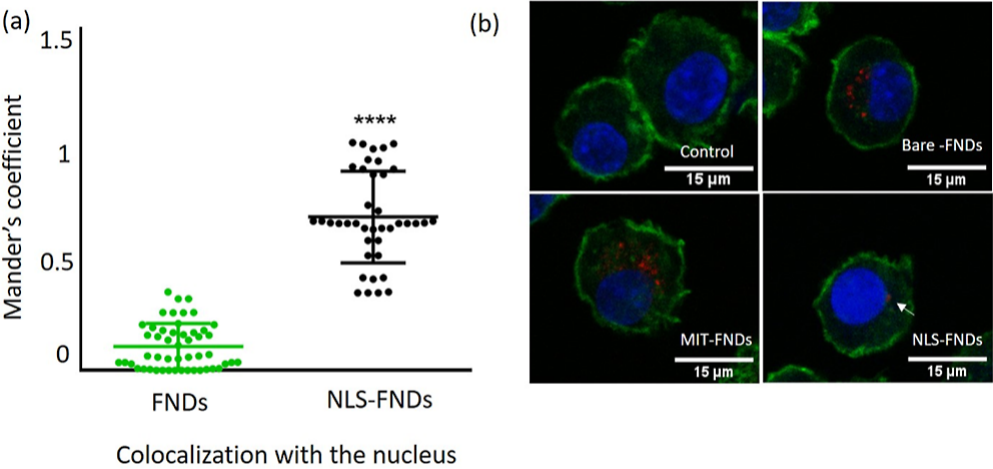
Colocalization of NLS-FNDs with the nucleus. (a) Manders’ colocalization coefficient of NLS-FNDs with the nucleus compared to FNDs. The scatter dot plot shows the standard deviations (unpaired *t*-test, ****P ≤ 0.0001). Experiments were repeated three independent times. (b) Confocal images of internalized FNDs, MIT-FNDs, and NLS-FNDs [blue: nuclei stained with DAPI, red: FND (with or without coatings), and green: FITC-phalloidin staining the actin filaments].

To confirm that NLS-FND is indeed targeted to the nucleus, we stained the nuclei and determined colocalization between NLS-FND and the nucleus. As a control, we also performed these experiments with bare FNDs (see Figure 2). There are also some particles that are localized at the nuclear surface in the FND group. This has also been observed in other cell types where FNDs tend to accumulate near the nuclear surface.^45^ Almost all particles in the NLS-FND group colocalize with the nucleus, allowing measurements on the nuclear surface. The Manders’ coefficient is used to quantify the degree of colocalization between fluorophores. Its range is between 0 and 1. Larger coefficient means stronger evidence of co-localization between fluorophores with the target organelles.^46,47^

**Figure 3:**
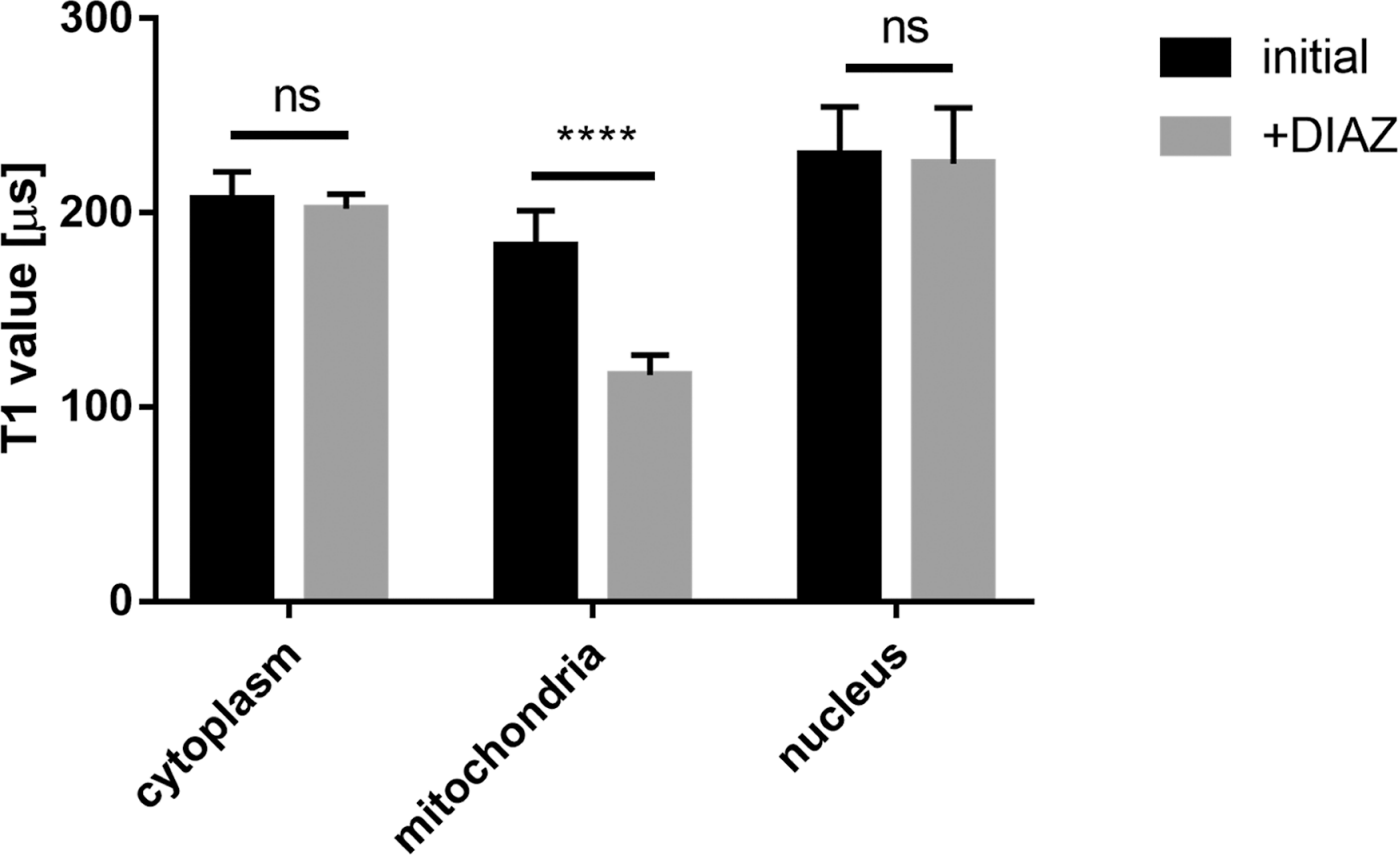
Control experiments to confirm localized free radical sensing. NLS-FNDs at the nucleus, FNDs in the cytosol, and MIT-FNDs at the mitochondria were exposed to diazoxide (50 μM) which triggers free radical generation at the mitochondria. The graphs are made based on six replicates of each variant, and error bars represent averages ± standard deviation (****P ≤ 0.0001).

Furthermore, we performed a control experiment to confirm that our T1 measurements are indeed localized to specific organelles. To this end, we used NLS-FNDs at the nucleus, FNDs in the cytosol, and MIT-FND at the mitochondria. Cells containing these particles were triggered with diazoxide which is known to trigger free radical generation in the mitochondria.^48^ The results of this experiment is shown in Figure 3. As expected, we observed a change in radical generation only for MIT-FNDs while the other groups did not respond to diazoxide.

Another important consideration is whether the particles retained their sensing performance. Since relaxometry is very local, it is important that the coating with targeting molecules does not prevent free radicals from getting close to the particles. We confirmed that this was still the case by test measurements where the coated and uncoated particles were exposed to Gd^3+^. Figure S2 confirms that it indeed still responds to changes in Gd^3+^ concentrations.

### Cell Viability

The metabolic activity of cells was tested with MTT assay to evaluate the effect of different concentrations of APAP as well as FNDs, MIT-FNDs, and NLS-FNDs. During this assay, MTT is converted to the purple formazan by mitochondrial reductase. Only metabolically active cells can convert MTT to formazan. It is important to note that the viability is generally considered equal to the control or if it is between 0.8 and 1.2 values. The results of the MTT assay are shown in the Figure S4. The cell viability after APAP treatment for 3, 6, and 9 h remain unchanged for all the tested concentrations. For the groups that were treated 18 h with APAP, we observed a reduction in viability in all groups exposed to different concentrations. These findings were consistent for groups containing bare FNDs, MIT-FNDs, and NLS-FNDs, which could be related to the accumulation of free radicals or other ROS after long-term exposure to APAP. While nanodiamonds have been shown to be excellently biocompatible in many different cell types or in vivo, it is important to confirm this for every new cell type and every new modification of particles.^42,49^ Thus, we included control groups where cells were only treated with bare FNDs, MIT-FNDs, or NLS-FNDs. As expected, none of these groups showed any reduction in cell viability.

### Measuring Stress Responses with Conventional Methods

#### Detection of ROS Generation by DCFH-DA Assay

**Figure 4:**
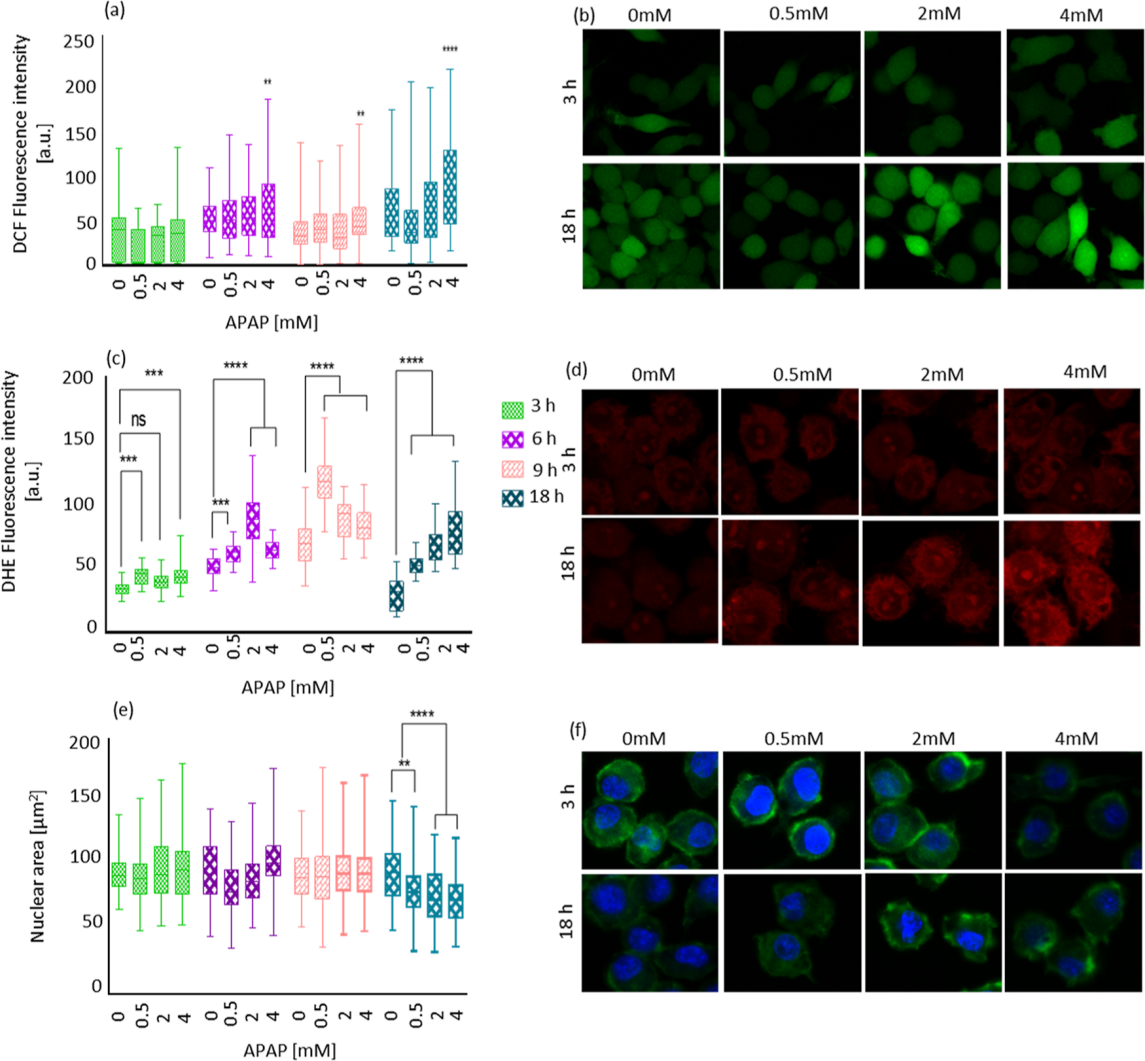
APAP induced ROS generation and total nuclear area on time points 3, 6, 9, and 18 h treated with 0, 0.5, 2, and 4 mM APAP. (a) DCFH-DA assay shows intracellular ROS generation after different time points of APAP treatment. (b) Representative confocal images from the DCF-stained macrophage J774A.1 cells. (c) Superoxide radical formation measured by the dihydro ethidium (DHE) assay. (d) Confocal images show the fluorescence intensity inside the cell caused by superoxide determined by DHE assay. (e) Total nuclear area. (f) Confocal images showing the reduction in total nucleus area after APAP treatment compared to the control group. Here, nuclei were stained with DAPI shown in blue, and actin fibers were stained with phalloidin-FITC shown in green. Scale bars are 15 μm. The data are shown by separated box and whisker plots with minimum and maximum values. Each experiment was repeated three independent times. Data were analyzed using a two-way ANOVA followed by a Tukey post hoc test. Statistical significance is indicated by *P ≤ 0.05, **P ≤ 0.01, and ****P ≤ 0.0001.

ROS production was tested by DCFDA assay. Using DCFH-DA as a fluorescent probe, we observed only significant increases in ROS production at the highest dose of APAP (4 mM, Figure 4a). This increase was evident for all time points. When exposed to the lower concentrations, the cells did not increase ROS production significantly. While this assay is widespread for its ease of use and availability, it has to be noted that it is not the most sensitive ROS assay available in the literature.^50^

The results from this method represent the average of a population of cells and the entire intracellular area.^47^ More precisely, it detects ROS including all kinds of free radicals and non-radical species such as for instance hydrogen peroxide, superoxide anions, or singlet oxygen species.^48^ Using DCFH-DA, we observed a high variability between the different cell groups (as shown in Figure 4a), which highlights the need for single-cell assays. Also, evident from Figure 4a is that there is no subcellular resolution. The reason is that DCF diffuses freely inside the cells^49^ during the whole incubation time. DHE, DCFH-DA, and other assays that are based on fluorescent probes also suffer from bleaching. As a result, it is important to take this bleaching into account. This also limits sequential measurements. FNDs on the other hand are stable and do not bleach.^51^

#### Measuring Superoxide by the DHE Assay

DHE freely permeates cell membranes and is used extensively to monitor superoxide production.^52^ It detects essentially superoxide radicals, is retained well by cells, and may even tolerate mild fixation.^53^ T1 relaxation time in mitochondria and the nucleus were evaluated with DHE assay. Using this assay, we detected superoxide formation after challenging the cells with 4, 2, and 0.5 mM APAP. As shown in Figure 4c, in almost all the groups, we observed an increase in superoxide formation.

#### Nuclear Area

Another way to assess toxicity is to monitor morphological changes. Due to the pronounced effect of APAP on the nucleus, we observed the changes in nuclear area. The results are shown in Figure 4c. At 3, 6, and 9 h, we did not observe any significant morphological changes. At the 18 h time point where we observed cell death for all the APAP concentrations, the nuclear area is also significantly reduced. This is expected since sub lethal concentrations normally do not lead to drastic changes in the morphology of the nucleus.

### Detection of APAP-Induced Intracellular Free Radical Generation with Relaxometry

The commonly accepted mechanism of APAP toxicity assumes that APAP induces free radical generation in the mitochondria. In addition, DNA damage in the nucleus has been reported as a result of APAP toxicity.^54^ To this end, we have decided to include measurements in the cytosol, the mitochondria, and the nucleus in this article.

#### Radical Generation in the Cytosol

After FND incubation, we took confocal images to confirm that FNDs were inside the cell and conducted relaxometry measurements. We treated cells with three different doses 0.5, 2, and 4 mM of APAP for 3, 6, 9, and 18 h and measured free radical generation via relaxometry. Decreasing T1 values indicates that the free radical concentration increased, while increasing T1 indicates a decreasing free radical load.^55^ T1 is equivalent to T1 in conventional MRI. However, since FNDs interact only with spins within a few nanometer, this method offers nanoscale resolution. Since contributing nuclear spins such as hydrogens are constant throughout the experiment and 3 orders of magnitude smaller, T1 gives a quantitative measure to assess radical generation in the particles surrounding. Figure 4a shows the free radical generation in the cytosol. We found that there are no significant changes in the free radical load in the cytosol in the first hours. Only at the highest concentrations of APAP and the longest incubation (18 h, 2 and 4 mM APAP), we observed an increase in radical formation. This radical formation is likely induced by cell death which occurs under these conditions. Cell death under these conditions was confirmed by MTT assay and confocal images showing reduced cell confluency (see Figures S4 and S6).

#### Radical Formation in the Mitochondria

**Figure 5:**
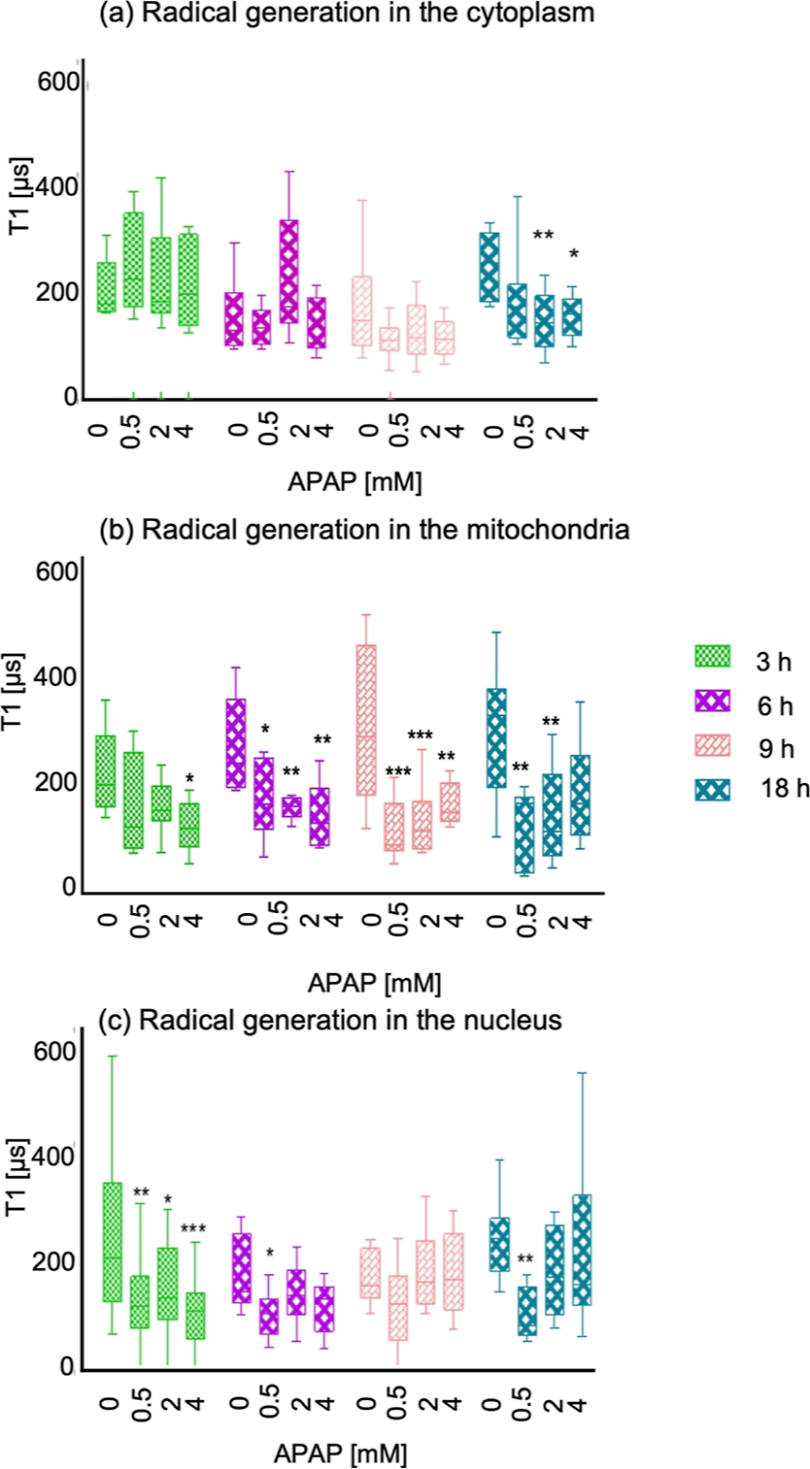
APAP-induced free radical response measured by relaxometry. (a) T1 relaxation in cells treated with different concentrations 0.5, 2, and 4 mM of APAP and different time points 3, 6, 9, and 18 h measured in the cytosol with FNDs. (b) Measurements on the mitochondrial surface using MIT-FNDs and (c) measurements on the nuclear surface using NLS-FNDs under the same conditions. The data are shown by separated box and whisker plots with minimum and maximum values. Each experiment was repeated three independent times. Data were analyzed using two-way ANOVA followed by Tukey post hoc test. Statistical significance is indicated by *P ≤ 0.05, **P ≤ 0.01, and ***P ≤ 0.001.

In contrast to the cytosol, in mitochondria free radical generation already occurred after 3 h of APAP administration (see Figure 5b) with the highest concentration of APAP (4 mM) and leads to a significant increase in radical formation. At time points 6, 9, and 18 h, however, we observed an increase in radical formation for all the concentrations, except for the 18 h 4 mM condition. This finding agrees with the literature. Al-Belooshi et al. demonstrated APAP-induced cellular toxicity in macrophages.^56^ They found increased ROS production in cells that were treated with APAP for 18 h compared to a control and the 2 h of treatment. They speculated that this is due to mitochondrial oxidative stress induced by NAPQI adduct formation. Compared to Al-Belooshi et al., we were able to see changes in the stress response earlier which might be related to free radicals being the first response to the adducts, higher sensitivity of our technique or mitochondria being the main source of the response, and the earliest responder organelle. At the earlier time points, we also observe a clear concentration dependency. In general, the higher the APAP concentration we used, the more pronounced free radical response we observed.

#### Detection of APAP-Induced Free Radical Generation in the Nucleus

Finally, we also investigated free radical generation on the nuclear surface. The results are shown in Figure 4c. In contrast to the cytosol or mitochondria, we observed free radical generation in the nucleus already at the 3 h time point with 0.5, 2, and 4 mM APAP, which represents a fast (3 h) and more sensitive (0.5 mM APAP) response when comparing with the other two evaluated compartments. This might explain findings in the literature where relatively low concentrations of APAP (0.1 mM) inhibited DNA synthesis within minutes in V79 Chinese hamster cells. RNA and protein synthesis in V79 cells are inhibited only after longer exposure (3 h) and considerably higher concentrations of APAP (3–10 mM).^57^

Furthermore, DNA strand breaks have been observed, and APAP inhibits both replicative DNA synthesis and DNA repair synthesis in vitro and in animal experimental models.^58^ While it has been assumed that these DNA alterations are caused by reactive species, we show here that radical species are indeed produced in or close to the nucleus. This finding is supported by reports on EPR spin trapping which has been employed to detect radical production in isolated rat liver nuclei on exposure.^59^ However, such studies required the use of spin traps and thus do not allow real-time measurements. Besides, those measurements were performed on large ensembles of cells rather than single cells.

Finally, our data show that radical formation also occurs in the nucleus or that radicals in significant amounts are accumulated there.

#### Controls without Cells

As a control, we also performed T1 measurements of the different FNDs exposed to different doses of APAP without cells. In the absence of cells and their stress response, we did not observe any significant differences from APAP alone (Figure S3).

### Comparing Methods for Free Radical Detection

While measurements with the conventional techniques were conducted on ensembles of cells, this is different in T1 measurements, where free radical measurements can be performed at a specific location inside one single cell. When comparing the different data in Figure 5a–c, it is clear that there are differences by location which are not accessible by the conventional techniques. Another difference with T1 measurements is that DCFH-DA or DHE assays reveal the history of a group of cells rather than the free radical concentration at a specific moment. It is also evident from Figure 4a that the DCFH-DA assay is less sensitive than our T1 measurements even though it measures a much larger sample. The reason might be that it does not allow local detection. It is also worth mentioning that due to the different detection mechanisms, relaxometry measurements have cross sensitivities that are different from the conventional probes.^50^ The conventional assays are influenced by non-paramagnetic ROS as well as certain enzymes or molecules which fluoresce in the same wavelength range. Relaxometry experiments, on the other hand, are influenced by spin labels or paramagnetic ions or extreme changes in pH.^30,40^ Additionally, there is an important difference in what exactly is measured. The conventional probes are not specific for paramagnetic molecules. The radicals are the molecules that have a free electron and are thus most aggressive. For this reason, this might be a good measure for the damage in the cell. This probably depends on the process of interest.

## Conclusions

We demonstrated the detection of radical generation with subcellular resolution in living macrophage J774A.1 cells. More specifically, we were able to show how cells respond to being challenged with APAP and where exactly the radical formation occurs. We found that while there is a relatively low radical load in the cytosol, there is an increase in radical formation in the mitochondria and the nuclear surface. Our measurements were able to detect the changes in the radical load early and in sub-lethal doses compared to conventional methods, relaxometry allows us to detect radicals specifically rather than all kinds of ROS. Furthermore, our technique allows monitoring the current state of the cells, whereas the conventional methods reveal the history of the sample. Our findings confirm the broadly accepted theory that the stress responses to APAP in macrophages happen primarily in the mitochondria and, to some extent, in the nucleus. While these findings are not surprising, the relaxometry method gives us a tool to directly measure the radical formation and determine the radical load in specific organelles, which highlights its potential for cellular toxicity evaluation in drug screenings.

## Supporting Information Available

Size distribution of diamond particles, sensitivity evaluation of functionalized particles with Gd^3+^, control experiments, cell viability test, confirmation of particle location, and confluency of the cell culture (PDF)
