# Supplementary material for: Intracellular Quantum Sensing of Free-Radical Generation Induced by Acetaminophen (APAP) in the Cytosol, in Mitochondria and the Nucleus of Macrophages
Source: ACS Sens. 2022 Nov 10;7(11):3326–34. doi: 10.1021/acssensors.2c01272 (PMC9706807; doi:10.1021/acssensors.2c01272)
Supplement: Supplementary file 1 — se2c01272_si_001.pdf [file se2c01272_si_001.pdf]

Supplementary

Intracellular quantum sensing of free radical generation induced by acetaminophen (APAP) in the cytosol, in mitochondria and the nucleus of macrophages

[1]Rokshana Sharmin, [1]Anggrek Citra Nusantara, [1]Linyan Nie, [1]Kaiqi Wu, [1]Arturo Elias Llumbet, [1]Willem Woudstra, Aldona Mzyk, [1]Romana Schirhagl*

1. Groningen University, University Medical Center Groningen, Dept. Biomedical Engineering, Antonius Deusinglaan 1, 9713 AV, Groningen, The Netherlands

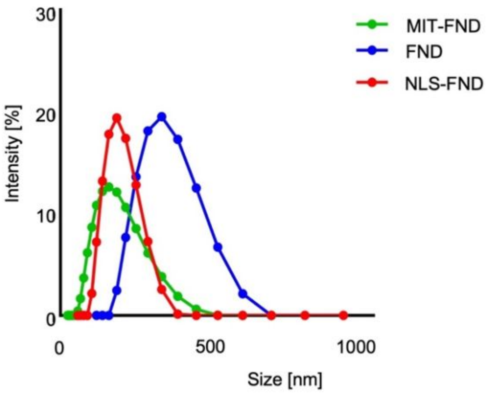

Supplementary Figure S1: Aggregation behavior of FND, NLS-FNDs and MIT-FND in DMEM medium. FNDs have an average size of 342 nm, while MIT-FNDs and NLS-FNDs average sizes are 290 and 190nm in DMEM medium. All measurements were performed three independent times at 37° C. The reason for the reduced size of the conjugated particles is that an existing protein layer on the particles prevents aggregation as shown in [26].

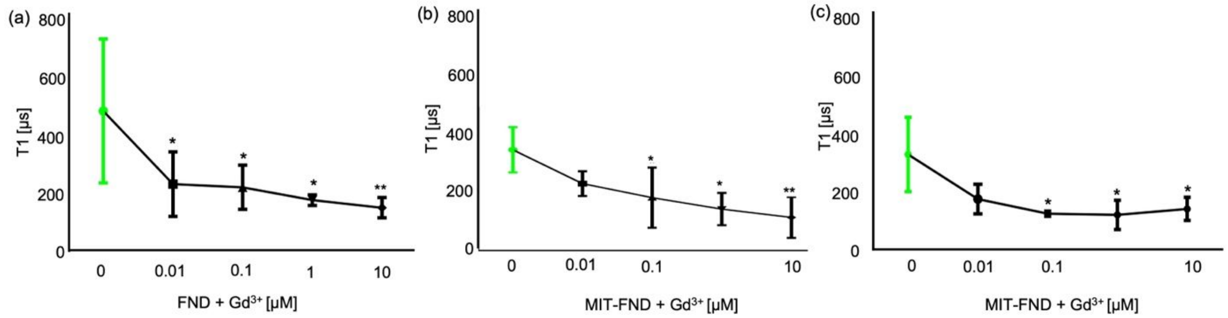

Supplementary Figure S2: Evaluation of spin sensing capacity of FNDs (a), MIT-FNDs (b) and NLS-FNDs (c) with differently concentrated $GdCl_3$. $GdCl_3$solutions were added to DMEM high glucose medium for measuring T1. The error bars represent the standard error of the mean from 4 particles. The data were analyzed using a one-way ANOVA followed by a Tukey post hoc test. Statistical significance is indicated by *P ≤0.05, **P ≤ 0.01.

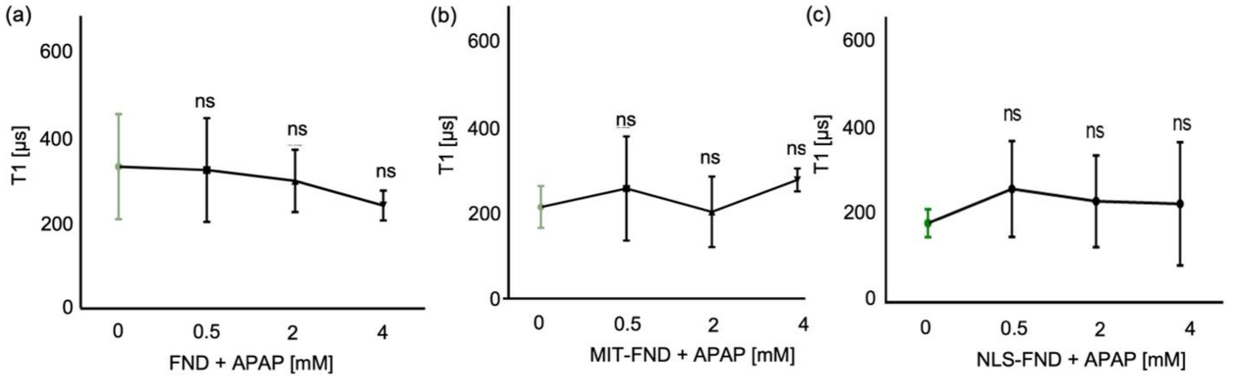

Supplementary Figure S3: Control experiments: FNDs (a), MIT-FNDs (b) and NLS-FNDs (c) without cells were exposed to APAP. T1 values were measured only in DMEM glucose medium by adding different concentrations of APAP. The error bars represent the standard error mean from 4 particles. Ns means non significant. The data were analyzed using a one-way ANOVA followed by a Tukey post hoc test. Statistical significance is indicated by ns > 0.05

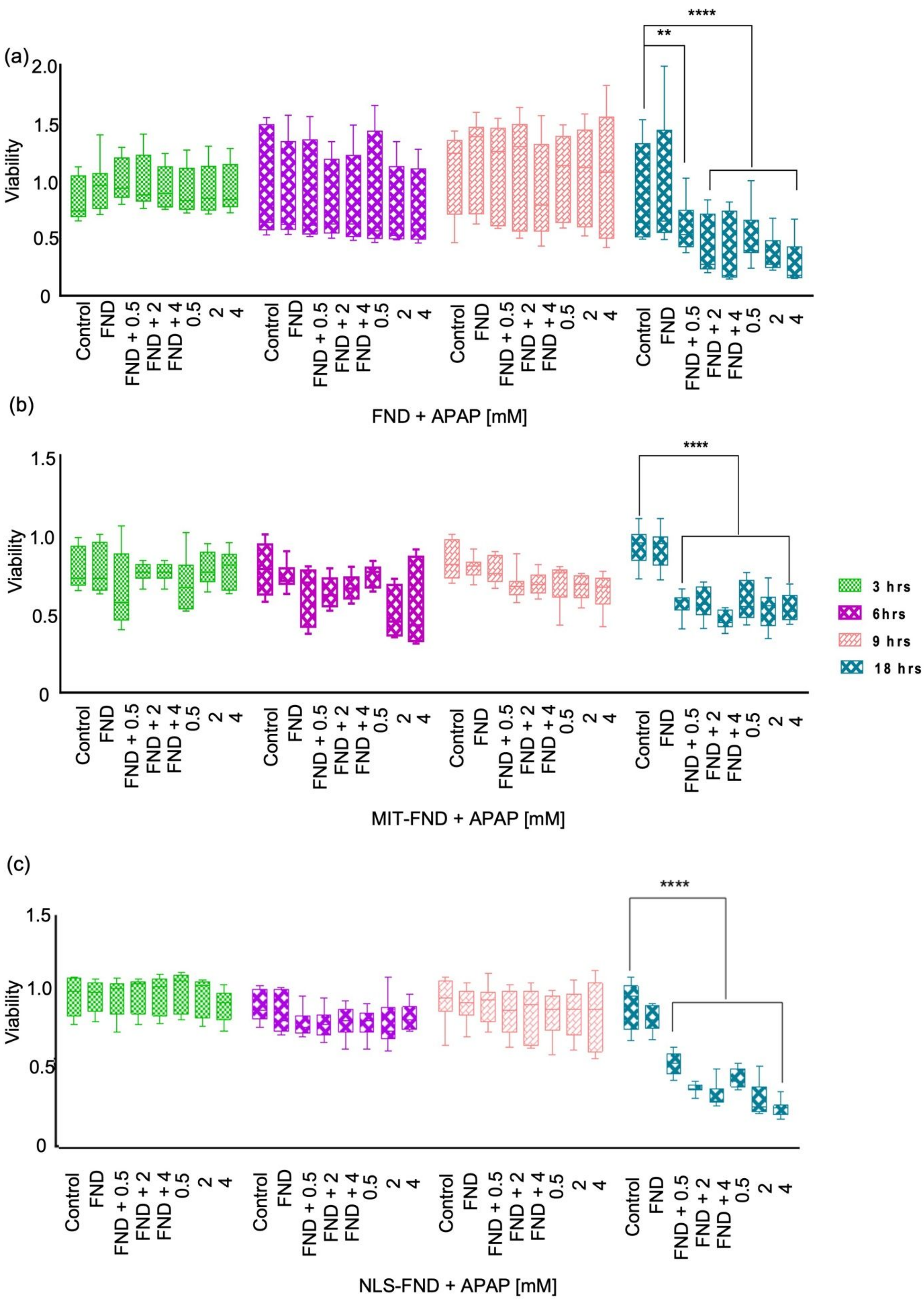

(a)
Viability
2.0
1.5
1.0
0.5
0
****
**
Control
FND
FND + 0.5
FND + 2
FND + 4
0.5
2
4
(b)
FND + APAP [mM]
Viability
1.5
1.0
0.5
0
****
MIT-FND + APAP [mM]
3 hrs
6hrs
9 hrs
18 hrs
(c)
Viability
1.5
1.0
0.5
0
****
NLS-FND + APAP [mM]

Supplementary Figure S4: cell viability after exposure of cells with FNDs (a), MIT-FNDs (b) and NLS-FNDs (c) in the presence of different concentrations of APAP. Cell viabilities were compared with the corresponding control groups. The data is shown by separated box and whisker plots with minimum and maximum values. Each experiment was repeated 3 independent times. Data were analyzed using two-way ANOVA followed by a Tukey post hoc test. Statistical significance is indicated by *P ≤0.05, **P ≤ 0.01, ****P≤0.000

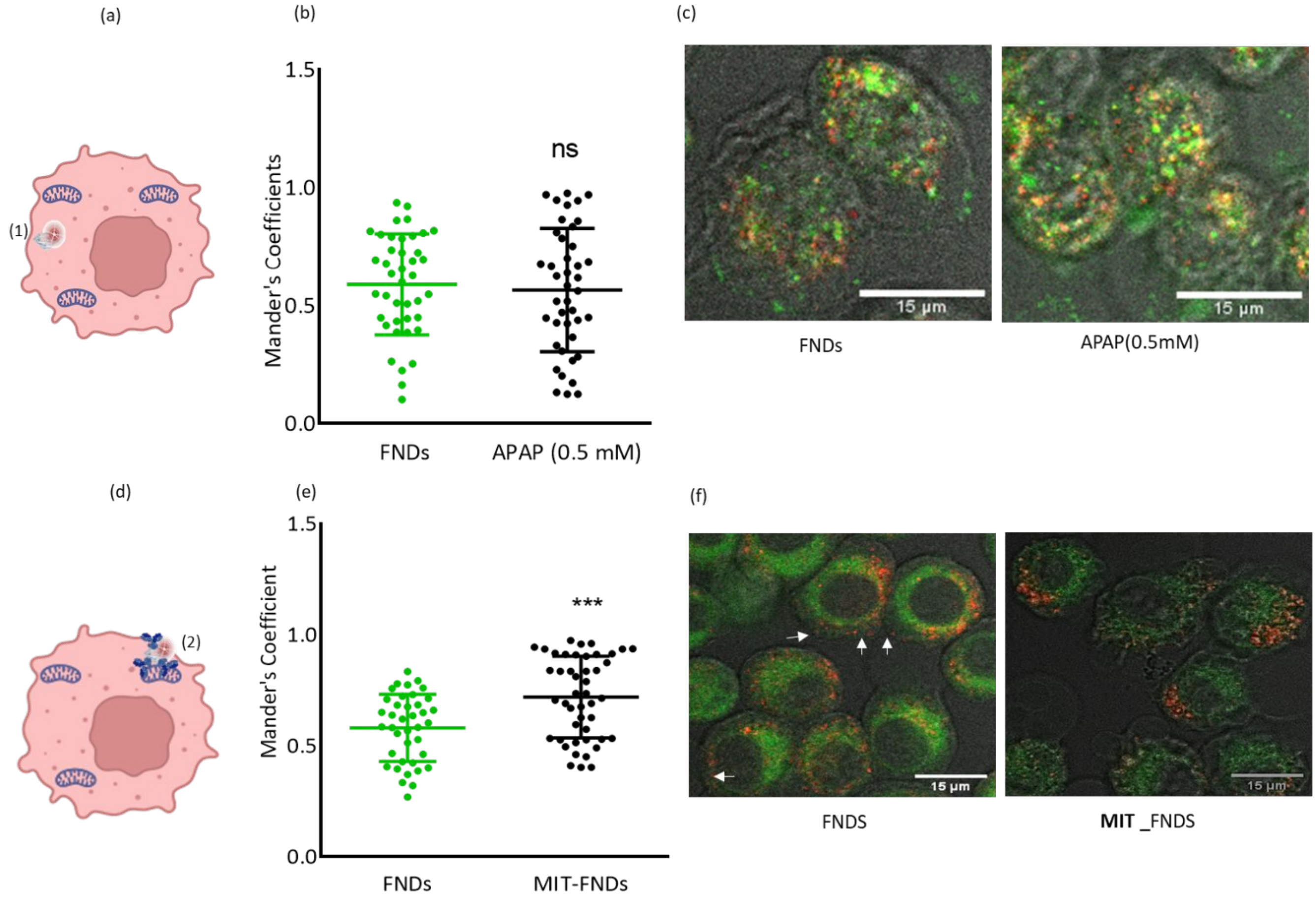

Supplementary Figure S5: Confirming the location of particles, (a) shows a schematic representation of the experiment (b) Colocalization of FNDS with vesicles (stained with calcein) determined by Manders' colocalization coefficient. As shown before [26] FNDs escape from endosomes and thus do not colocalize with calcein. We also show that the location does not change in the groups treated with APAP (0.5mM) for 18h. (c) Confocal images showing the FNDs (red) escaped from endosomes (vesicles stained in green). (d) is a schematic representation of the experiment. (e) shows the colocalization of MIT-FNDs and FNDs with mitochondria analyzed by using the Mander's colocalization coefficient. (f) Representative confocal images showing FND and MIT-FNDs (red) and mitochondria (green) stained with Mito Tracker green that specifically stains mitochondria. Scatter dot plots show the standard deviations. The scatter dot plots show

the standard deviations (unpaired t-test, ***p ≤ 0.001). Experiments were repeated 3 independent times.

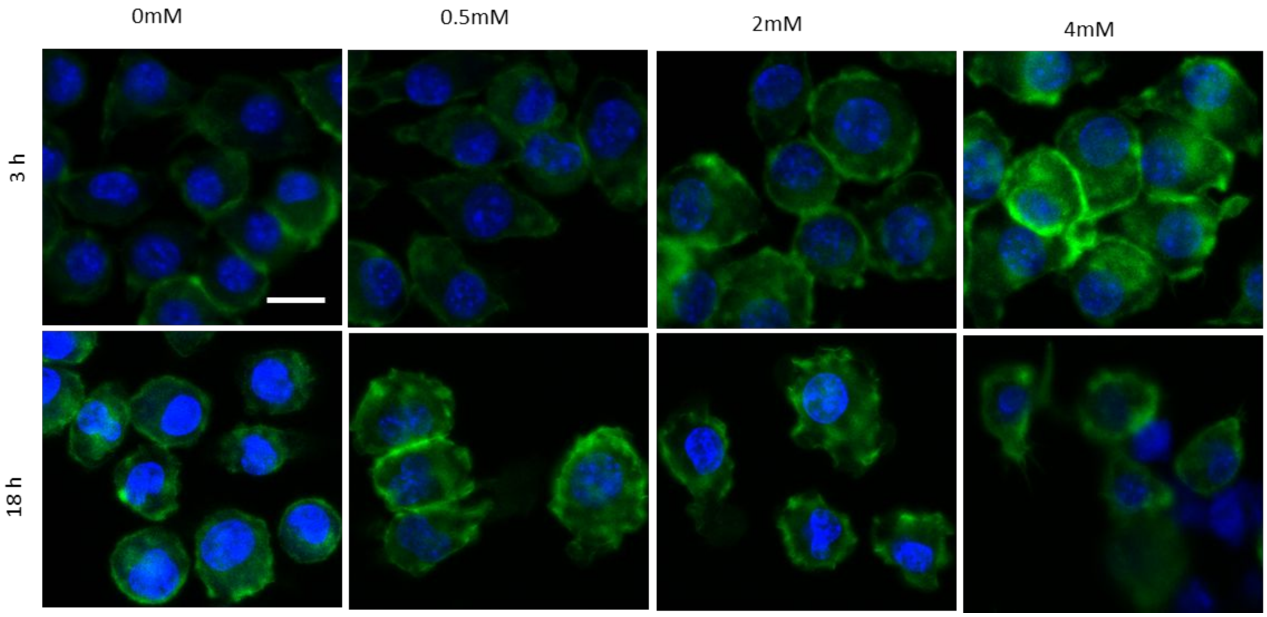

Supplementary Figure S6: Confocal images show the confluency loss after 18 h of APAP treatment compared to 3 h of APAP treatment. Most of the cells from the 18 h treatment groups were washed out during the following steps of staining whereas cells from 3 h APAP treatment groups were retained and maintained the confluency. Scale bar is 15 µm.
